# “What is language for us?”: Community-based Anishinaabemowin language planning using TEK-nology

**DOI:** 10.1007/s10993-023-09656-5

**Published:** 2023-05-12

**Authors:** Paul J. Meighan

**Affiliations:** Department of Integrated Studies in Education, McGill University, McTavish Street, Montréal, Québec Canada

**Keywords:** Anishinaabemowin, Technology, Language policy, Language planning, Indigenous language revitalization, Language reclamation

## Abstract

Language planning and policy (LPP), as a field of research, emerged to solve the “problem” of multilingualism in newly independent nation-states. LPP’s principal emphasis was the reproduction of one-state, one-language policies. Indigenous languages were systematically erased through top-down, colonial medium-of-instruction policies, such as in Canadian residential schools. To this day, ideologies and policies still privilege dominant classes and languages at the expense of Indigenous and minoritized groups and languages. To prevent further erasure and marginalization, work is required at multiple levels. There is growing consensus that top-down, government-led LPP must occur alongside community-led, bottom-up LPP. One shared and common goal for Indigenous language reclamation and revitalization initiatives across the globe is to promote intergenerational language transmission in the home, the community, and beyond. The affordances of digital and online technologies are also being explored to foster more self-determined virtual communities of practice. Following an Indigenous research paradigm, this paper introduces the *TEK-nology* (Traditional Ecological Knowledge [TEK] and technology) pilot project in the Canadian context. *TEK-nology* is an immersive, community-led, and technology-enabled Indigenous language acquisition approach to support Anishinaabemowin language revitalization and reclamation. The *TEK-nology* pilot project is an example of bottom-up, community-based language planning (CBLP) where Indigenous community members are the language-related decision-makers. This paper demonstrates that Indigenous-led, praxis-driven CBLP, using *TEK-nology*, can support Anishinaabemowin language revitalization and reclamation and more equitable, self-determined LPP. The CBLP *TEK-nology* project has implications for status and acquisition language planning; culturally responsive LPP methodologies; and federal, provincial, territorial, and family language policy.

## Introduction

Language planning activities are not new; they have been carried out by humans since time immemorial, from everyday communicative choices to contemporary forms of policymaking (McCarty, 2018a; Wright, 2016). Language planning and policy (LPP), as a field of research, first originated in the 1950s and 1960s to “solve” the “problem” of multilingualism in newly independent nation-states (Fishman, 1968; Spolsky, 2018). The common view among western sociolinguists during this time was that linguistic diversity was problematic for national development and “unity” (May, 2006; Ricento, 2000). LPP was a solution to the problem of multilingualism and multiculturalism and its principal emphasis was on promoting and reproducing “unifying” one-state, one-language policies (Phyak, 2021; Spolsky, 2018). Indigenous languages were systematically erased through top-down, colonial medium-of-instruction policies, such as residential schools in what is now known as Canada, where the “aim of education is to destroy the Indian” (Davin, 1879). To this day, ideologies and policies still privilege dominant classes and languages at the expense of Indigenous and minoritized groups and languages (Fishman, 1991, 2001; May, 2021; Phyak, 2021).

More recently, in response to historical and structural inequalities due to colonialism and imperialism (i.e., the “historical-structural approach”; Tollefson, 1991), a critical sociocultural paradigm of LPP has emerged (McCarty & Warhol, 2011). This critical paradigm recognizes LPP as “the complex of practices, ideologies, attitudes, and formal and informal mechanisms that influence people’s language choices in profound and pervasive everyday ways” (McCarty, 2011, p. vii). Critical LPP research underscores researcher positionality, an ongoing critical and self-reflective examination of the researcher(s) relationship with those involved in the research (Lin, 2015; Tollefson, 2006). The critical perspective strives for equity and social justice and recognizes that, although LPP has been weaponized by dominant groups to maintain systems of privilege, it can also be transformative (McCarty, 2018a; Skutnabb-Kangas, 2000; Spolsky, 2017; Tollefson, 1991). There is growing consensus that successful top-down, government-led LPP must occur alongside community-led, bottom-up LPP (Ricento & Hornberger, 1996). Hornberger (1999) and May (1999, 2006) assert that Indigenous language revitalization (ILR) initiatives can only succeed if the community is significantly involved in planning and development.

One shared and common goal for ILR initiatives across the globe is to promote intergenerational language transmission in the home, the community, and beyond (Fishman, 1991, 2001; Hinton, 2013; Leonard, 2017). Drawing on critical sociocultural approaches to LPP (McCarty, 2018b; Tollefson, 2006) and Leonard’s (2017) language reclamation framework, this paper will introduce the *TEK-nology* (Traditional Ecological Knowledge [TEK] and technology) pilot project in the Canadian context. *TEK-nology* is an immersive, community-led, and technology-enabled Indigenous Language Acquisition (ILA) approach to support Anishinaabemowin Language Revitalization and Reclamation (ALRR). The *TEK-nology* pilot project is an example of online community-based language planning (CBLP; McCarty, 2018a). CBLP is bottom-up, grassroots, and emphasizes the agency and autonomy of Indigenous communities in language-related decision making (Hornberger, 1999; Lewis et al., 2016; McCarty, 2018a).

The *TEK-nology* project is collectively led by community participants and the researcher to support community-led language revitalization and cultural reclamation processes and to center their expertise and knowledges. The purpose of the *TEK-nology* project is to explore relationships between community-led ILA, place-based knowledge, and technology in the Canadian context while responding to policy calls for technology to be culturally appropriate and rooted in Indigenous worldviews (Government of Canada, 2018; Truth and Reconciliation Commission of Canada, 2015). This paper seeks to: (1) expand the critical sociocultural paradigm of LPP (McCarty & Warhol, 2011); (2) exemplify further praxis-driven and technology-enabled CBLP research (McCarty, 2018a); and (3) illustrate how the engagement of language minoritized communities in LPP (Phyak, 2021) and Indigenous-led CBLP can inform more equitable language policy through “culturally grounded contexts of praxis” (May, 2021, p. 49).

## The impact of inequitable language planning and policy on Indigenous and minoritized language communities

Language dominance, shift, or “death” is neither natural nor unavoidable (Dorian, 1998; Skutnabb-Kangas & Dunbar, 2010). As May (2012) remarks, “language loss is not only, perhaps not even primarily, a linguistic issue – it has much more to do with power, prejudice, (unequal) competition and, in many cases, overt discrimination and subordination” (p. 4). Language policy has enabled dominant language groups to maintain nation-state power and hegemony at the expense of Indigenous and minoritized language communities (Tollefson, 1991), where “subordinate languages are despised languages” (Grillo, 2009, p. 174).

### Ongoing threats to Indigenous cultural, linguistic, and epistemic heritages

Indigenous communities on Turtle Island (or what is also known as North and Central America) and across the globe have been multilingual and multicultural since time immemorial (Canagarajah, 2005; May, 2021; McIvor & McCarty, 2017). Multilingualism is not new; it was commonplace worldwide prior to colonial and imperial expansion and “ideologies of contempt” towards Indigenous languages (Dorian, 1998; Grillo, 2009). Multilingualism was the normal, not the “problem” before the imagined one-nation, one-language community associated with the western nation-state system (May, 2012; McIvor & McCarty, 2017; Phyak, 2021). Multilingualism in Indigenous communities in Turtle Island precedes “new” present-day sociolinguistic superdiversity (May, 2021). Multilingual practices in early contact between Indigenous communities and Europeans already “included the use of multilingual interpreters, lingua francas and trade jargons, and mixed languages” (Patrick, 2012, p. 35). Understanding that Indigenous multilingualisms and multiculturalisms have existed prior to colonialism and imperialism, so-called “new” superdiversity in Global North, and continue to thrive beyond is a vital starting point to address lingering colonial frontier logics, Eurocentric ideologies and epistemologies, and monolithic assumptions of cultures and languages that relegate Indigenous language communities to the “past” (Daniels & Sterzuk, 2021).

Indigenous communities worldwide continue to face threats to their linguistic and epistemic heritage with the unabated spread of dominant colonial languages and global monocultures, such as English and the neoliberal, imperialistic worldview (Battiste, 2013; Phillipson, 1992; Phyak & Sharma, 2021; Skutnabb-Kangas, 2000). Two-thirds of the world’s 7000–7500 languages are Indigenous languages; one-third of those are experiencing language loss (Lewis et al., 2016), and “as many as 90% are predicted to fall silent by the end of the century” (McCarty, 2018a, p. 23). Some people may assume that language loss is “normal”. However, language *shift* and *loss* differ from language *change*. McCarty and Nicholas (2014) remark, “all languages change through time as a result of language-internal processes and as their speakers interact with other speech communities and cultural changes require new linguistic forms” (McCarty & Nicholas, 2014, p. 107). In contrast, language shift is “concretely mirrored in the concomitant *destruction* of intimacy, family, and community, via national and international…intrusions” (Fishman, 1991, p. 4). This destruction leads to:

Community-wide [language] shift, which occurs when the social structures supporting intergenerational language transmission break down, often as a result of violent dominant-subordinate encounters and the coerced abandonment of ancestral mother tongues. When external forces interact with internal ones, they can produce feelings of linguistic ambivalence and shame, furthering the cycle of language loss. (McCarty, 2018b, p. 356)

Nation-state medium-of-instruction policies have long been a driving force of language shift (Tollefson & Tsui, 2004). Mainstream education and colonial, restrictive policies have attempted to “‘erase and replace’ linguistically encoded knowledges and cultural identifications with those associated with dominant-class ideologies, values, and practices” (McCarty, 2018b, p. 356). These policies have led to educational and socioeconomic inequities for Indigenous peoples, such as poverty, low rates of educational attainment, and teen suicide (Castagno & Brayboy, 2008). In Canada, Indigenous linguistic and cultural heritage has been compromised either through overt force and genocide, as in residential schools (Truth and Reconciliation Commission of Canada, 2015), or in more covert forms, such as present-day monocultural, monolingual school environments. While the destructive role and devastating impacts of residential schools is becoming increasingly recognized (Hanson, Gamez, & Manuel, 2020),

English and French remain the primary medium of instruction of Indigenous students in most schools across the country and attendance is compulsory. Even in schools with Indigenous language programs, students still do most of their learning, speaking, thinking, and functioning in English or French rather than in their ancestral language. (Fontaine et al., 2017, p. 8)

### Preventing erasure through Indigenous-led and community-based language planning

Indigenous languages barely receive “ideological and implementational space” (Hornberger, 2005) in mainstream western education. To prevent further erasure and marginalization, work is required at multiple levels. Extensive scholarship underscores that top-down implementation must take place with community-led, bottom-up LPP (King, 2001; Lin & Yudaw, 2016; McCarty, 2018a; Meek, 2011; Ricento & Hornberger, 1996; Shohamy, 2006; Spolsky, 2014). On Turtle Island, for example, “one-size-fits-all” approaches do not work given the great cultural and linguistic diversity and vast geographic span of Indigenous peoples and languages. According to the most recent Census data, there are 169 Indigenous languages in the United States (Siebens & Julian, 2011) and more than 70 in Canada (Statistics Canada, 2017). Research also demonstrates that Indigenous language reclamation and revitalization are most effective when they are community-driven and responsive to local contexts and needs (Leonard, 2012; May, 1999; McCarty, 2018a; Ricento & Hornberger, 1996). Conversational knowledge of an Indigenous language can save Indigenous lives, raise community capacity, and foster deep social, emotional, and spiritual wellbeing (Hallett et al., 2007; Kirmayer et al., 2016). Youth suicide rates effectively drop to zero with conversational knowledge of an Indigenous language (Ball et al., 2013; Hallett et al., 2007).

Local, bottom-up, grassroots initiatives are leading the way for Indigenous language reclamation and revitalization, which is also growing as an academic discipline (McIvor & McCarty, 2017). In recent years, proficient and fluent speakers are emerging from Indigenous community-led and self-determined initiatives. Many of these tend to be predominantly or completely immersive in the language and have a strong culture- and land-based component to support intergenerational language transmission, address the privileging of dominant, colonial languages and knowledges in mainstream education and policy (Meighan, 2022c), and counteract extreme language shift (McCarty, 2018b, 2021; Phyak, 2021). Indigenous community-based language planning (CBLP) is a key example and is characterized by the agency of local people in language-related decision making (Lewis et al., 2016; McCarty, 2018a). CBLP often begins with a small group or even an individual. These efforts by individuals and families led to changes in national and state-level language policies. McCarty (2018a) elaborates, “it was a small group of Indigenous parents and elders who established the first Kōhanga Reo and Pūnana Leo in the early 1980s, at a time when Māori and Hawaiian were predicted to ‘die’” (p. 373). In Finland, Saami community members implemented a year-long course with classes and cultural activities to support Aanaar Saami revitalization (Olthuis et al., 2021). In Nepal, a Limbu community youth organization sought to ensure the Limbu language is taught in school (Phyak, 2019). And in the North American context, Wôpanâak (Wampanoag) Native American tribal citizens revived their language, which had not been spoken in more than 150 years. The community-based Wôpanâak Language Reclamation Project (WLRP) was led by the efforts of a single tribal citizen, jessie little doe baird, and today offers classes, immersion camps, and a language nest preschool (little doe baird, 2013).

The affordances of digital and online technologies are also being explored to foster more self-determined CBLP, to enable Elders and speakers to reach more learners, and to bolster existing local initiatives (Herman et al., 2020; Olthuis et al., 2021; Olthuis & Gerstenberger, 2019; Toth, Smith, & Giroux, 2018). For example, the WLRP highlighted above also offers Kun8seeh, an online community where Wampanoag language learners can “engage with your language whenever you want, from wherever you want” (www.kun8seeh.com/). Many more Indigenous communities, especially during the COVID-19 pandemic, have been using digital and online technologies to sustain and continue important community-based initiatives (McIvor et al., 2020; McIvor, Chew, & Stacey, 2020). Examples of community and land-based immersion classes and programs that went online due to COVID-19 are the nêhiyawak Language Experience for Cree learners (Daniels et al., 2022), or Eshki-Nishnaabemjig for Anishinaabemowin learners (Anishinabek News, 2020). Tollefson (2017) points out, “language planning may take place in schools and other institutions, in families and workplaces, or in any social group—including virtual communities—in which verbal communication takes place” (p. 2).

As the literature demonstrates, to be more equitable LPP needs to be community-, culture-, and context- specific. To further explore the potential of CBLP with “virtual communities” (Tollefson, 2017), I introduce the *TEK-nology* pilot project for ALRR. *TEK-nology* is an immersive, community-led, and technology-enabled ILA approach. The article will address three research questions.

RQ1) What does technology-enabled CBLP look like in practice? What are its possibilities, tensions, and challenges?

RQ2) How do Indigenous community members conceptualize culturally and environmentally responsive LPP, research, and education?

RQ3) How can Indigenous-led CBLP address systemic inequities and the marginalization of Indigenous peoples in LPP, research, and education?

## Methodology

### Positionality

Is mise Pòl Miadhachàin-Chiblow. ’S e Gàidheal a th’ annam. Rugadh agus thogadh mi ann an Glaschu, Alba. My name is Paul Meighan-Chiblow. I’m a Scottish Gael. I was born and raised in Glasgow, Scotland.

My research focuses on multilingual and multicultural education, Indigenous language revitalization, and language policy. My experiences as a Gàidheal (Scottish Gael) growing up in Glaschu (Glasgow) inform my work. I was raised by my mother who is from Dalabrog (Daliburgh), in the north-western island of Uibhist a Deas (South Uist) in na h-Eileanean Siar (Western Isles). I remember hearing Gàidhlig (Scottish Gaelic) all the time around my fluent speaking grandmother, who was a core of our family. However, Gàidhlig, an endangered Indigenous language in Alba (Scotland) with approximately 57,000 speakers, was not available to me in the educational system. Gàidhlig and Gaelic culture were almost eradicated due to many factors, such as the forced eviction of the Gàidheil (Gaels) from their traditional homes and lands during the Highland Clearances in the mid-18th to -19th centuries and the destruction of centuries-old Gaelic clan-based society after the Battle of Culloden in 1746 by British government and imperial forces (e.g., Hunter, 2014; MacKinnon, 2017). In more recent times, members of my own family and older generations recall being beaten for speaking the language in classrooms. An example is the maide crochaidh (the “hanging” or “punishment” stick) that children passed along to those who were caught speaking Gàidhlig (MacKinnon, 2019). Moreover, Gàidhlig, spoken for more than 1500 years in Alba, is still not recognized as an official language in the United Kingdom. The multi-generational and psychological impacts of the trauma associated with the repression of Gàidhlig and Gaelic culture linger to this day and have been driving factors for language shift, “loss”, socioeconomic and sociopolitical inequities, and the near destruction of family and community intergenerational language transmission in Alba (e.g., Smith, 1982; Ó Giollagáin, 2020). McFadyen and Sandilands (2021) elaborate,

The ongoing legacy of this coloniality of power is destructive in a myriad of ways. In the *Gàidhealtachd* the effects of clearance are still felt, with a fragile economy, rural housing crisis and the decline of the Gaelic language. In his essay, *Real People in a Real Place*, Iain Crichton Smith spoke of historical ‘interior colonisation’ alongside a growing materialism which, he believed, had left Gaels in a cultural milieu increasingly ‘empty and without substance’…such a view resonates with…perspectives made by writers and scholars of indigenous peoples across the globe. This is not to suggest or promote an equivalence here between the experience of the descendants of enslaved people and others who experienced colonisation by modern, imperial states; rather, such perspectives describe symptoms of human-ecological disconnect, alienation and loss of meaning – an indicator of just how far our human psyche and culture has become divorced from our natural environments. (p. 163)

As a direct result of deliberate processes of covert and overt linguistic eradication, family land dispossession, the role of the educational system, and internalized deficit ideologies about the “value” of Gàidhlig, I do not speak my language fluently *yet*. I am currently on a Gàidhlig reclamation journey as an adult learner, which also forms part of my ongoing self-decolonization process (see also Meighan, 2022b).

**Fig. 1:**
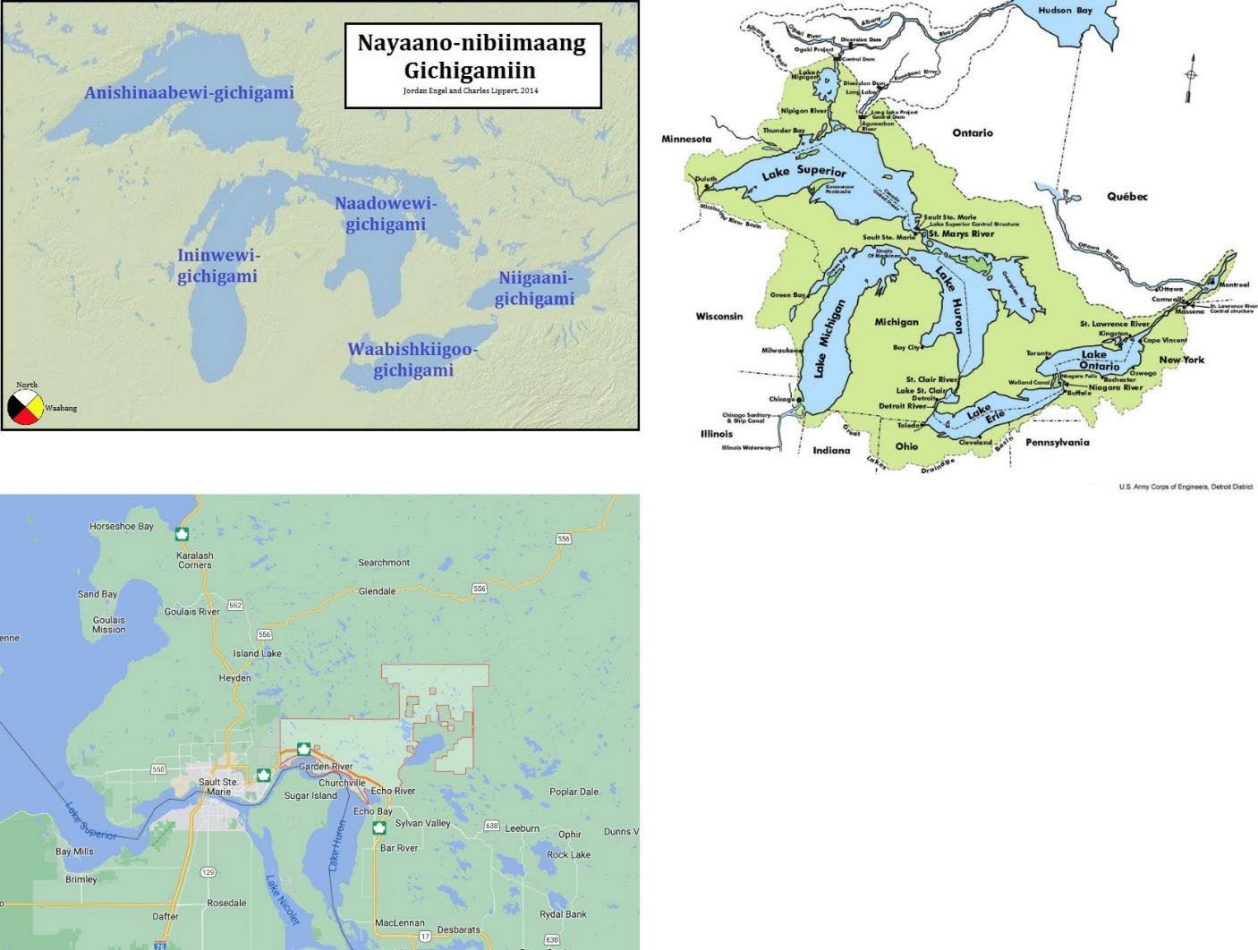
*Map of Nayaano-nibiimaang Gichigamiin (Great Lakes Region) and Ketegaunseebee (Garden River First Nation)*

My motivation for equitable education, language policy, and language revitalization has continued to grow after meeting my Anishinaabe Ojibwe husband in Glaschu, Alba in 2015. After marrying there, I immigrated to Turtle Island together with him in 2016. Since then, I have learned more about the devastating impacts of colonialism on the Indigenous Peoples of Turtle Island from him and from discussions with my Anishinaabe family. These experiences have led to my current research, which focuses on the role of technology for the maintenance, reclamation, and revitalization of endangered and Indigenous languages. This research project explores CBLP using the *TEK-nology* approach with participants from my Anishinaabe family’s community in Ketegaunseebee (Garden River First Nation) in the Great Lakes Region of Turtle Island (see Fig. 1). As a (re)searcher and family member who is not Indigenous to Turtle Island nor from Ketegaunseebee, I respectfully follow Anishinaabe protocols and methodologies on this project.

### Research context and methods

Ketegaunseebee has a population of 3,264 members registered under the Indian Act, according to latest statistics. 1,350 members are resident on the band’s reserve, while 1,914 members live off reserve (Crown-Indigenous Relations and Northern Affairs Canada, 2022). According to the latest Census data, 115 members—10.2%—of the on-reserve population report knowledge of an Indigenous language, which refers to “whether the person can conduct a conversation in the language” (Statistics Canada, 2016). In the latest Census, 97.3% of the on-reserve population report speaking English most often at home (Statistics Canada, 2016).

The *TEK-nology* pilot project^1^ took place over 13 weeks between September-December 2021 during the COVID-19 pandemic. There were several phases: an online autoethnographic self-examination journal; offering Asemaa (Tobacco) in gratitude; individual semi-structured conversations with participants (N = 7) who later formed a Language Revitalization Committee (LRC); one LRC sharing group and three LRC focus groups; the creation of three 3-minute *TEK-nology* language learning videos; and a final online survey (see Table 1). Due to COVID-19, research involving participants was conducted remotely using online (Zoom) and digital technologies (laptop, cellphone, and camcorder). Asemaa should be offered in person when seeking Elder guidance and/or assistance in line with Anishinaabe protocols (Wilson & Restoule, 2010). Due to COVID-19, I offered Asemaa in gratitude to the land on which I was located, Tkaronto (Toronto), before speaking online with participants.

**Table 1:** Research participants and procedures

Research participants	Procedures
Researcher (N = 1)	Ongoing self-decolonization Online autoethnographic self-examination journal *TEK-nology* video editing
Community members (N = 8) > 18 years (N = 7) < 18 years (N = 1)	*Language Revitalization Committee (LRC)* (N = 7 > 18 years) 1 x Individual semi-structured conversation (45 min) 3 x focus groups (1.5 h each) 1 x sharing group (1.5 h) *TEK-nology videos* (N = 3 LRC members + N = 1 < 18 years) 3 × 3-minute language video co-creations
Community members (N = 8)	1 × 10-question online survey (3 questions on 4-point Likert scale and 7 open-ended questions)

The *TEK-nology* pilot project was rooted in an Indigenous Anishinaabe paradigm, Mino-bimaadiziwin (The Good Life). I followed an Anishinaabe community-led, decolonizing, participatory methodological framework, Biskaabiiyang, or “Return to Ourselves” (Geniusz, 2009). Biskaabiiyang begins with the researcher decolonizing themselves to conduct meaningful research with the Indigenous community (Geniusz, 2009). As a methodology for my ongoing self-decolonization process, I followed Dùthchas (which I loosely translate as Ancestral Bonds). Dùthchas is an intrinsic part of the sealladh a’ Ghàidheil (Gaelic worldview) and is derived from the Gàidhlig word “dú / dùth”, meaning “earth” or “land” (MacKinnon & Brennan, 2012). Dùthchas, as a Scottish Gaelic ontology and methodology, stresses the interconnectedness of people, land, culture, and an ecological balance among all entities, human and more than human (Meighan, 2022b). Following Dùthchas, and prior to starting the *TEK-nology* project, I began to learn and reclaim my endangered Indigenous language, Gàidhlig, to connect more with my mother culture and resist colonialism and language oppression. Prior to COVID-19, I participated in Anishinaabe ceremony in my family’s community, and during COVID-19, I took an Anishinaabemowin for Absolute Beginners online course with Elder and Anishinaabek Nation Language Commissioner Barbara Nolan, who later joined the project. Biskaabiiyang grounds me in community-led, Anishinaabe protocols, values, and ethical practice while Dùthchas guides me for a respectful and non-appropriative (self)-decolonizing research journey informed by my own lived experiences. These decolonizing methodologies respond to Wilson’s (2001) call to be “answerable to all your relations when you are doing research” (p. 177).

**Fig. 2:**
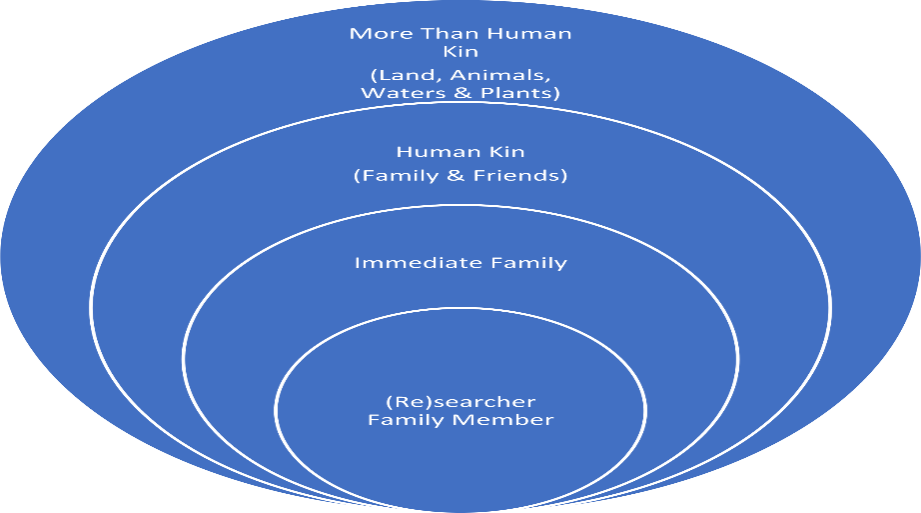
Kincentric and relational approach to sampling

**Fig. 3:**
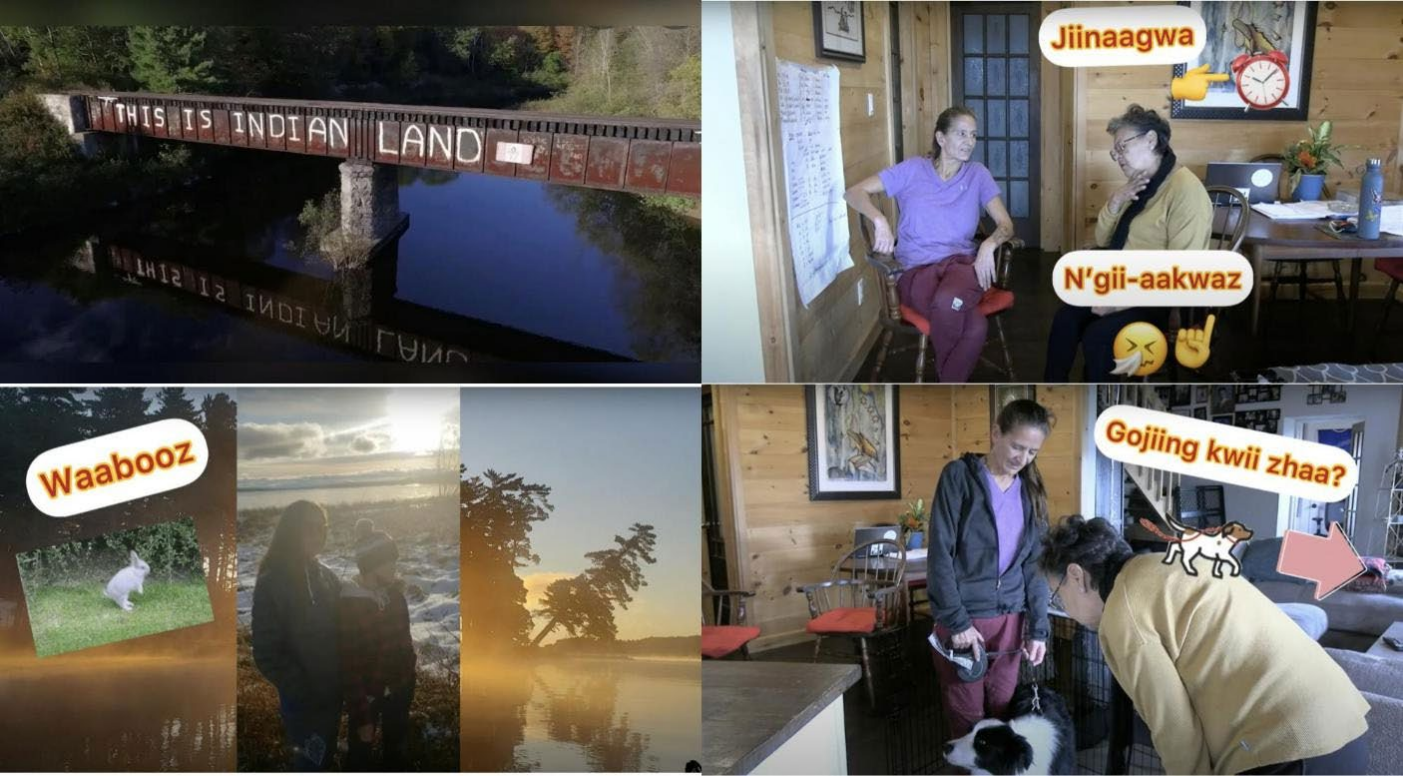
TEK-nology Indigenous language acquisition video screenshots

Participants were selected through purposive and snowball sampling (Patton, 2015) from family and family friends from my Anishinaabe family’s community in Ketegaunseebee^2^. I designed and implemented a kincentric and relational approach (Fig. 2) for participant selection to (1) continue deepening and respecting existing relationships, and (2) understand factors within those relationships that may have influenced intergenerational ALRR. Together, we, the community participants and I, formed the LRC. I conducted individual semi-structured conversations with LRC members, using the Conversational Method (Kovach, 2010) which gathers knowledge in relational dialogue with the “deep purpose of sharing story” (p. 40). We had three LRC focus groups to generate ideas, themes, and content for videos. We discussed relationships between Anishinaabemowin, the land, and technology. In our sharing group, we shared what language education means and is for the community. After this, we co-created three 3-minute immersive and conversational Anishinaabemowin *TEK-nology* videos between the fluent speaking Elder and a fellow LRC member (see Fig. 3). The videos were filmed by LRC members themselves in Ketegaunseebee. At the end of the project, the videos were shown to participants at a *TEK-nology* video screening on Zoom for collective feedback and approval. The videos are now hosted on a public LRC YouTube channel (https://www.youtube.com/playlist?list=PL-uUUEW1KLsu-1SKs-Ixd8MQGnLGa88wP) in accordance with participant wishes. Participants were invited to respond to an online survey for feedback on the pilot project and the co-creation process. LRC members were also invited to provide feedback on this article and others about the project (see Meighan, 2022a, for further discussion).

To interpret and analyze the knowledge generated during the project, I employed qualitative Anishinaabek data analysis (Chiblow, 2021), alongside inductive reflexive thematic analysis (RTA) (Braun & Clarke, 2021). Inductive RTA worked alongside Anishinaabek analysis as part of the greater Indigenous theoretical framework and paradigm to ensure validity and accountability to both Indigenous and non-Indigenous (academic) communities.

## Analysis

I identified three main themes during analysis: (1) *Good research*; (2) *Language reclamation for community capacity building*; (3) *Strengths in community-based education.* I report examples of these in a storied manner that is reflective of our conversations to maintain the integrity of the participants’ insights and knowledge (Kovach, 2009, 2010). As Hermes & Kawai’ae’a (2014) point out, “the data are in the stories” (p. 304).

### Good research

At the beginning of the project, LRC members and I had an individual semi-structured conversation to better understand what CBLP and LPP research should look like. Participants stressed that good community-based research is inclusive and holistic. Good research should be beneficial and accessible to the participants and the wider community. According to Joseph Belleau, Indigenous education teacher and family friend,

Good research is when the material is providing the context of lived experiences, whether it be social, emotional, or physical aspects. Hands-on always, for myself, is easier to comprehend and grasp. Some people are so used to reading stuff and having that kind of understanding. But, for someone like myself, having pictures, images, tactile stuff in front of me…Providing that as research and then letting myself get ahold of that learning is key. (Individual conversation, 6/9/21)

Elder Barbara Nolan, who has decades of experience teaching and designing curricula for Anishinaabemowin language courses in the community and beyond, highlighted researcher reflexivity and relational research objectives:

I think it all depends on what your goal is. What do you expect to get out of the research? You have to work towards that. I always find that when you’re researching, you have to get at the core of your question: is it going to reflect on your goal? That’s what I think is good research. Your sources of the research have to be credible. Credible individuals or credible material that is relative to your research. (Individual conversation, 8/9/21)

Jayce Chiblow, Toolkit Action Lead for Indigenous Climate Action and the researcher’s sister-in-law, underscored the importance of Indigenous-led research:

I think research should be Indigenous-led. And when I say led, there can be non-Indigenous researchers. But I think when it comes to design and inclusion and overall oversight of the project or the research that’s going on, it should be Indigenous-led and include more than just one type of person. I think there should be a variety, including all genders, including all age groups…having a wide variety of input so that the design itself and the execution of the research is done in a good way. (Individual conversation, 3/9/21)

Doing research in a good way was similarly stressed by Dr. Susan Bell Chiblow, Assistant Professor in the School of Environmental Sciences at the University of Guelph and Jayce Chiblow’s mother:

Good research is research that actually benefits people, not just people, but also benefits all living beings. That’s what I think good research is. From an Anishinaabek perspective, good research is living life, following Mino Bimaadziwin, living a good life. Because as Anishinaabe people, we didn’t necessarily ‘research’, we were always searching for knowledge. So that lifelong journey, I think it’s part of good research. (Individual conversation, 3/9/21)

In an effort to conduct good research, I kept a reflexive self-decolonizing/examination journal to document my (re)search learning journey. I wrote about things such as what decolonization and decolonizing research means to me; challenges and tensions; and my language learning journey in Anishinaabemowin and reclamation journey in Gàidhlig. Below is an excerpt from my first online entry about the research project and participant selection as part of the kincentric and relational methodological approach I designed (see also Fig. 2; Meighan, 2022b):

Today, I’m at the point where I’m about to send out invitations to potential Language Revitalization Committee (LRC) and *TEK-nology* video participants. I’m very mindful that I would like to embark on this project in my married family’s community in the most relational way possible, and I think a good way of doing that is by starting with myself and radiating outwards.

Me.

Close immediate family.

Extended family.

Relational human kin (close family friends that I know or who have been recommended to me).

Relational more than human kin (the land).

I’m imagining this working like a concentric circle (insert image when I get to drafting it) … It is my sincere hope that I can embark on this project in a good way…I’m not from the community where I will be working myself, so I would like to start with people I know, to include people who are close to my immediate relations in the project. I want to centre the people who know the land and their community and the process of reclaiming their language at the heart. They will guide the process…I think the thing I would like most is to be transparent to myself and also to anyone else going forward. And this journal will be helpful (I hope) in tracking that journey and the experiences I have. (August 16, 2021)

In our LRC focus groups, we discussed how technology could support language transmission and be implemented in a beneficial and good way. We agreed where the *TEK-nology* videos should be kept (public YouTube); what knowledge or content is shared (natural, everyday conversations); and which teaching and learning approach (language immersion) would be implemented. We decided the videos should be short and done in a natural, fun, conversational manner to engage learners of all ages and learning styles. To co-create the *TEK-nology* videos, we sought to incorporate an immersive, concept-based ILA approach Elder Barbara Nolan suggested. This approach involves, “not resorting to grammar…you teach a concept, all in the language…maybe you have a picture of something, and you’re going to put the ball on the table. You’re teaching locative words, but you’re not telling people these are locative words” (B. Nolan, individual conversation, 8/9/21). In the anonymized online exit survey, in response to Question 8 “How do you feel about the language learning/transmission process on this project compared to other experiences you have had?”, 3 out of 6 respondents commented on the benefits of technology-enabled and Indigenous-led CBLP:

“I really enjoyed the interaction with everyone when I was available to attend. I think it provided great insight for everyone to share their experiences with one another and learn from that.” (Online survey, 31/1/2022)

“I feel this is the way to go on language transmission - virtually as a lot of people can be reached.” (Online survey, 31/12/2021)

“This was much more community based and focused on a variety of levels. Most of the processes I’ve experienced included a main focus on grammar and technical knowledge where this project allowed us to focus on what we wanted, and included immersion!” (Online survey, 20/12/21)

### Language reclamation for community capacity building

In our individual conversations, focus, and sharing groups, we shared ideas for raising engagement for community-based language learning within and beyond the *TEK-nology* project. Debra Nolan, Elder Barbara’s niece, emphasized, “With the hustle and bustle of everyday, it just needs to take priority in your life” (Individual conversation, 8/9/21). Joseph Belleau commented:

This is based on my own experience. When I talk to my parents, or my aunts and uncles in regard to language, no one really speaks Ojibwe…They have taken French…It’s not a top priority for a lot of people. I think we have to make it a priority to retain the language, and to change that frame of thought in regard to what is being Anishinaabemowin. What is language for us? What things can we grow and flourish within our community? Because we do have a lot of opportunities to see a lot of these things into fruition. What are the steps we need to take regarding community engagement? Community education regarding language? I think we have everything. (Focus Group 1, 26/9/21)

Jayce Chiblow, who works in British Columbia, shared what language strategies she has seen in Indigenous communities there and in Rankin, one of the communities in Batchewana First Nation, near Ketegaunseebee. She also mentioned High Bank, a popular landmark with views over Ketegaunseebee:

I was driving down the highway here, it’s called the Sea to Sky Highway. The Squamish folks are really great at reclaiming their language. They have all these signs…[and] I think it’s in Rankin, they use stop signs, like slow down or children playing signs. But what that made me think of is using our language for place names. Because on that highway they had all the signs in their language for the name of that area, and every two minutes you drive there’s another one. If we want to start collective community learning, a good way would be to start reclaiming our place names. What’s High Bank’s name? I don’t know, but we all know High Bank. What about the mouth of the river...we must have the name for that. Starting with smaller things, like places that we use, would be a great way for collective community learning and to inspire people. (Focus Group 1, 26/9/21)

On the topic of future community-based and -led initiatives, Elder Barbara Nolan underscored the influence of dominant and deficit language ideologies on ALRR:

There’s still some people that say, why do you want to learn the language?...That state of thinking has got to disappear. We have to look at the other side. If we lose our language, we are going to lose more than language. We’re going to lose what’s in that language. There are some explanations in some of the words in our language that mean a great deal more than the English translation for that word. But that won’t come until we create speakers and then the speakers, the newly created speakers, can access that type of education by looking at the words. The other thing is Pierre Elliott Trudeau. In one of his talks when our Chiefs went to see him, he said, if you do not have your first language, then you have no business coming to see me about whatever…And that is so true, because if we lose our language, if it completely disappears…what’s going to hold us together as Nishnawbe people when we don’t have a language?...We cannot lose our language. That’s a bottom line. (Focus Group 1, 26/9/21)

Debra Nolan suggested ways in which ILA and ALRR could be fostered and incentivized within the community and beyond. She gave the example of her daughter and fellow LRC member, Sydney Nolan, who took French immersion until Grade 12:

Why isn’t Anishinaabemowin a requirement to hire? I think it would push more people to acquire their language…If there is a more of a need and a want for those higher positions… I sent her [Sydney Nolan] to a French immersion because I was thinking, what would get her the best jobs and what would help her…And it did. She has French as a second language, and she did get hired because she is bilingual. I think if she acquires Anishinaabemowin, that would help her as well. (Focus Group 1, 26/9/21)

During our focus groups for the *TEK-nology* language videos, immersion was identified as a key language acquisition strategy. Elder Barbara remarked, “if you want to acquire language and become a speaker, immersion is the way to go” (Focus Group 3, 28/11/21). She elaborated on the success of French immersion in Canada and its implications for CBLP and ALRR in Ketegaunseebee:

In the mid 60s, I think it was, the government said we’re going to have a bilingual country, English and French… So, the people in Saint Lambert, Québec were saying our kids are not coming out of Grade 8 speaking French. We have to do something about that. There were two professors from McGill University who went and helped them with the very first immersion, French as immersion class, kindergarten class…They wanted their kids to get these jobs…all these government jobs that require you to be bilingual. Why can’t us Nishnawbe people do that? But we have to create immersion, kindergarten…All those classes are going to be taught in the language…I don’t see a fully Anishinaabemowin immersion school yet. (Focus Group 1, 26/9/21)

Looking beyond bottom-up CBLP, LPP strategies at a macro-level were also discussed. Dr. Bell Chiblow commented on the success of Māori language immersion and revitalization and its implications for future ALRR initiatives:

The Māori did a 10-year language strategy and now most of the country knows how to speak that language. But the challenge when you look at Turtle Island is there’s so many different language groups. I’ve also heard old people say, don’t worry about the dialect. For instance, Anishinaabemowin has different dialects, and they say don’t worry about that dialect. Just learn it from someone and the understanding of the differences will then come. So, trying to think about the government, the Truth and Reconciliation Committee said one of their recommendations about language is the government committing to a language strategy, developing a Language Commission, or having a Language Commission. But how do you put a 10-year language strategy into each kind of pocket of community? For instance, I think if we did it from Robinson Huron Treaty territory... if we did it in territories as opposed to communities, then it would be more successful. But is the government willing to commit the funding to those type of activities? (Individual conversation, 3/9/21)

### Strengths in community-based education

Participants shared ideas and strategies on what community-driven and -based education can and should be. Karen Bell, Garden River First Nation Band Councilor for the Educational Programs portfolio, who was taught Anishinaabemowin by Elder Barbara Nolan in elementary school in the 1970s, spoke about the role of culturally responsive language education in creating strong individuals:

Just teaching students English is not sufficient. If you have a predominant classroom of Indigenous children, then you better be speaking that language or trying to engage them in speaking the language, because that’s when you learn who you are. That’s when you learn where you’re from, and that’s where you learn, from the bottom of your heart and your mind, to connect with each other. This is where you start feeling really good about yourself and your confidence, and all those other things that build strong individuals. (Individual conversation, 7/8/21)

Participants underscored the strong connections between Anishinaabemowin, community, culture, and identity. Joseph Belleau remarked, “identity in itself is very key in regard to revitalization, not only of language, but of us as Anishinaabe” (Focus Group 1, 26/9/21). Sydney Nolan commented, “language, to me, is defining who you are” (Individual conversation, 16/9/21). In our sharing group, in response to “In what ways can learning Anishinaabemowin build strength in the community?” Elder Barbara Nolan, a residential school survivor, shared,

I think it builds a sense of pride, too. It fills up your identity because that’s what we lost at the residential school. We lost the ones who were there for a long period of time, lost their language, they were forbidden. We were forbidden to speak our language, so the ones who were there much longer than I was didn’t speak the language when they come out of there after ten years. And they lost their identity. So, this helps strengthen one’s identity. You know, when you’re learning the language, and you get the piece of yourself back. (Sharing group, 28/11/21)

Land-based, hands-on, experiential learning was identified as key for ALRR and for community education. Jayce Chiblow shared, “There’s concepts that come from the land. There’s teachings in our language that come from the land” (Individual conversation, 3/9/21). Debra Nolan echoed these words:

As Anishinaabe People, we believe everything has a spirit and carries a spirit. As do I, and I have my name. Each of these plants and medicines that belong to the land have a spirit and a name, and they should be directed as such…whether it be on the land, or Nibi (water), they each have their name…I know the best way I learn is hands-on. I have to see it. I’m more visual. Say [for example], Nibi. Then you hold the water. Nibi…Nibi. (Individual conversation, 8/9/21).

Sydney Nolan, Debra Nolan’s daughter, further described the relationship between Anishinaabemowin and the land:

Most of my teachings come from the people around me, or even the plants around me, the animals around me, my teachers are everyone around me…Once you learn the language…most of it has different parts of the land tied into the word. It’s very significant. A lot of our language would not be there if it wasn’t for the land, because that’s where we’re most rooted. (Individual conversation, 16/9/21)

On the topic of ILA and ALRR, participants shared context- and community- specific factors that can support language learning, motivation, and progress. Dr. Susan Bell Chiblow remarked:

I think listening, understanding it first is just as important as being able to speak it. Somebody also said that a baby isn’t born knowing how to speak a language…Looking at it from that perspective helps me not be so hard on myself if I don’t understand the language. (Individual conversation, 3/9/21)

Sydney Nolan shared an interaction she had with her great-aunt, Elder Barbara, while she was learning Anishinaabemowin:

Once I asked her, so how do you spell that? She goes, well, no, write it as you think it sounds because the grammar does not worry. She goes, as long as you know what you’re saying, as long as you can pronounce it, that is fine. So, that’s where it made me think, I was like, whoa! That is a totally different way of teaching, or that way I was taught for French. It really made me think about, in school with French, it was grammar, verbs, and then pronouncing, hearing, listening. But with her, it really made me understand more and feel like I was progressing without having to be grammatically correct. That’s where I realized...Progress, should it really be categorized? (Individual conversation, 16/9/21)

Elder Barbara Nolan elaborated on her immersive, concept-based teaching approach with young learners in the community:

I speak only the language. I don’t translate anything I say to them…I have fun. That’s a one way of passing on a language… I don’t force them to speak the language, they will speak the language when they are comfortable…One time, I was helping get them ready to go outside to play…This one little guy walks by me, and I said to that little guy, ‘Aapiish e-zhaayin?’ (Where are you going?). That little guy turns around, looks at me, and he says, ‘Gojiing!’ (Outside!). He answered me in the language. And my heart was full. (Focus Group 1, 26/9/21)

Several respondents shared in the online survey how the immersive, concept-based language videos we co-created on our CBLP *TEK-nology* pilot project could support ALRR.

“They can help learners learn new words.” (Online survey, 24/1/22)

“I learned some new words from the videos and it has me trying to learn more words to work on my conversational abilities.” (Online survey, 20/12/21).

“I learned new words/phrases.” (Online survey, 19/21/21).

“They are about everyday conversations. Plus they are fun.” (Online survey, 19/12/21).

Respondents also commented on how we could branch out more into the wider community using technology-enabled spaces in the future:

“I learned in this project that all of us have the same goals for wanting language to be present within our community.” (Online survey, 31/1/2022)

“I’d like to see more, and hopefully some mobilization in GR [Garden River]” (Online survey, 20/12/21)

“More videos - maybe even a GRFN [Garden River First Nation] website dedicated to videos in the language” (Online survey, 19/12/21)

## Discussion

The *TEK-nology* pilot project explored and conducted praxis-driven CBLP using a self-determined, technology-enabled language acquisition approach rooted in Indigenous educational philosophies and worldviews (Blair et al., 2018; Government of Canada, 2019; McCarty, 2018a; Truth and Reconciliation Commission of Canada, 2015). The research questions asked what technology-enabled CBLP looks like in practice; how Indigenous community members conceptualize culturally and environmentally responsive LPP and education; and how Indigenous-led CBLP could address systemic inequities in LPP and education. This section will discuss key takeaways from the analysis in response to the research questions: (1) *Indigenous-led, beneficial research*; (2) *Immersive, place-based Anishinaabe language education policy;* (3) *Strength-based and self-determined community-based language planning*.

### Indigenous-led, beneficial research

The *TEK-nology* pilot project is an example of CBLP (McCarty, 2018a) conducted entirely through digital and online technologies with implications for status and acquisition language planning (Cooper, 1990; Kaplan & Baldouf, 1997, 2003) and “methodological rich points” in LPP research (Hornberger, 2015). The analysis indicates that technology is a beneficial medium for CBLP where participants can “share their experiences with one another and learn from that” (Online survey respondent, 31/1/2022). The *TEK-nology* language videos, now hosted on public YouTube, made the research co-creation more accessible to participants and potential future community learners. As one online survey respondent remarked, “I feel this is the way to go on language transmission - virtually as a lot of people can be reached” (Online survey respondent, 31/12/2021).

The conversations, interactions, and video co-creations with the LRC on the *TEK-nology* pilot project underscore that CBLP for ALRR should be Indigenous-led and beneficial to the community and beyond (May, 1999, 2006; McCarty, 2018a). Indigenous scholarship stresses the necessity of researcher positionality, or “self-location” prior to embarking on research *with* or *by* Indigenous communities (Absolon, 2011; Kovach, 2009; McGregor et al., 2018; Riddell et al., 2017). Absolon (2011) underscores that “location does matter. People want to know who you are, what you are doing, and why” (p. 73). Elder Barbara invited the question, “What do you expect to get out of the research?” (B. Nolan, 2021, personal communication). Self-location identifies power differentials in LPP and “prompts awareness of the extractive tendencies of (western) research” (Kovach, 2009, p. 112). Acknowledging subjectivity through researcher positionality and self-location is fundamental for future critical LPP work that deems to be beneficial and transformative (Lin, 2015). It is therefore essential that researchers position themselves and follow community- and culturally- specific protocols for good research, beneficial LPP, and praxis-oriented CBLP (McCarty, 2018a) to: (1) avoid the western “helicopter approach” (Hall et al., 2015) where researchers arrive in marginalized and Indigenous communities, collect data, and rarely ever return; (2) set the stage for equitable research methods and “researchers-in-relation” (Kovach, 2009), which privilege Indigenous ways of knowing and being within LPP and research more broadly; and (3) expand the critical sociocultural paradigm of LPP (McCarty & Warhol, 2011).

As a family member who is not Indigenous to Turtle Island, I self-located and positioned myself in relation to the research (see Methodology) and followed an Anishinaabe research paradigm—Mino Bimaadiziwin (The Good Life)—and decolonizing methodologies. Mino Bimaadiziwin was highlighted as integral to good research by one of the participants (S. Bell Chiblow, 2021, personal communication). The Anishinaabe paradigm helped ensure the research followed ethical parameters, such as the 6 Rs of Indigenous research: respect, responsibility, relevance, reciprocity, relationship, and refusal (McGregor et al., 2018) and Ownership, Control, Access, and Possession (OCAP) standards (First Nations Information Governance Centre, 2014). OCAP standards assert that Indigenous communities maintain control over research and are recognized as knowledge holders. The decolonizing methodologies (Biskaabiiyaang and Dùthchas) I followed enabled me to respect and be accountable to existing relationships within my Anishinaabeg family’s community (relational accountability; Reo, 2019; Wilson, 2001) and take an “engaged approach” to LPP which, “focuses on trust-building with the researched communities and on creating an environment where participants can openly discuss multiple and contested language issues (e.g. sociopolitical, educational, and cultural) in education” (Phyak, 2021, p. 222). I kept a reflexive self-decolonizing and examination journal (Moeke-Pickering et al., 2006) to document my (re)search learning journey. Strega and Brown (2015) state, “reflexivity–a recognition that the researcher is not separate from but exists in relationship with what s/he is trying to understand–is a core component of ethical research practice” (p. 8). This journal helps me activate inward knowledge as much as possible (Kovach, 2009). The CBLP *TEK-nology* research co-creation process, resulting articles, and the ultimate evaluation and assessment of the language videos were also subject to LRC and Elder peer review and member checking, which served as a form of validity. The LRC also functioned to ensure community participants were language-related decision-makers (Ricento & Hornberger, 1996; Lewis et al., 2016; McCarty, 2018a), “where this project allowed us to focus on what we wanted” (Online survey respondent, 20/12/21).

### Immersive, place-based Anishinaabe language education policy

The CBLP *TEK-nology* pilot project highlights the importance of making language learning a priority. The analysis indicates that technology-enabled CBLP enabled a space, or virtual language planning community (Tollefson, 2017) for “ideological clarification” (Kroskrity, 2004), where participants shared strategies and thoughts on Anishinaabe language education. Participants indicated that there is a need to change the “frame of thought” (Belleau, 2021, personal communication) and “state of thinking” (B. Nolan, 2021, personal communication) which privilege dominant languages, such as English or French, and influence local language-making decisions and language shift (Phyak, 2021). Phyak (2021) elaborates, “it is important to engage language-minoritized communities in dialoguing about the impacts of dominant language policies in their communities” (p. 230). All participants stressed the strengths of the relationships between Anishinaabemowin and the land. In contrast to acquiring or learning dominant or non-endangered languages, ILA is place-based and a process that is inseparable from the land, culture, community, and worldview (Chiblow & Meighan, 2021; Hammine, 2020). Intergenerational language *and* knowledge transmission in the local family-school-community nexus is crucial for ILA and CBLP (Corntassel, 2008; McCarty, 2018a, 2021). Indigenous languages transmit highly specialized place-based knowledges, such as Traditional Ecological Knowledge (TEK) and unique medicinal knowledge (Absolon, 2011; Cámara-Leret & Bascompte, 2021; Geniusz, 2009). Indigenous-language immersion (ILI) is the most effective approach to ensure transmission of place- and land- based knowledge alongside language (McCarty, 2018b, 2021). The fluent speaking Elder on the project underscored that “immersion is the way to go” (B. Nolan, 2021, personal communication). Equally as important in Indigenous language education is contesting and counteracting dominant language hierarchies of prestige where Indigenous languages can be relegated to informal language domains and viewed as *only* “marginal” or “local” languages (Liddicoat, 2013). It is necessary for Indigenous languages to be present in formal language domains, such as education, government, and media *alongside* intergenerational transmission in the family and community (May, 2000). Reclaiming place names and making street signs in Anishinaabemowin was given as a suggestion for “collective community learning and to inspire people” (J. Chiblow, 2021, personal communication) to acquire the language. Reclaiming place names and making street signs could raise the status of Indigenous and minoritized languages through “prestige planning” so that “members of the targeted speech community develop a positive attitude toward it” (Kamwangamalu, 2016, p. 158). Participants shared additional strategies to raise the status of the language within the community and beyond, such as the requirement of Anishinaabemowin for jobs (D. Nolan, 2021, personal communication) and a fully immersive Anishinaabemowin school, inspired by the success of parent and community pressure for French immersion (B. Nolan, 2021, personal communication). As Meades, Pine, and Broad (2019), who researched the regional labour market for Anishinaabemowin, remark, “Indigenous language training is not a frill, but an integral part of meeting the labour force demands of Indigenous communities” (p. 59).

The CBLP *TEK-nology* pilot project has implications for status and acquisition language planning (Cooper, 1990; Kaplan & Baldouf, 1997, 2003) and more equitable and culturally responsive LPP and education that centres Indigenous Peoples, languages, and their communities. CBLP using *TEK-nology* expands the critical sociocultural paradigm of LPP (McCarty & Warhol, 2011); exemplifies further praxis-driven, technology-enabled CBLP research (McCarty, 2018a); and illustrates how the engagement of Indigenous and language minoritized communities in LPP (Phyak, 2021) can inform more equitable language policy through “culturally grounded contexts of praxis” (May, 2021). The analysis illustrates that Anishinaabe community members on the *TEK-nology* pilot project are the language-related planners and decision-makers (Lewis et al., 2016; McCarty, 2018a). Anishinaabe community members decide what language is and means for them and center their own community needs rather than externally defined or set goals, such as grammatical fluency (Leonard, 2017) or a digitally “thriving status” (Kornai, 2013).

**Fig. 4:**
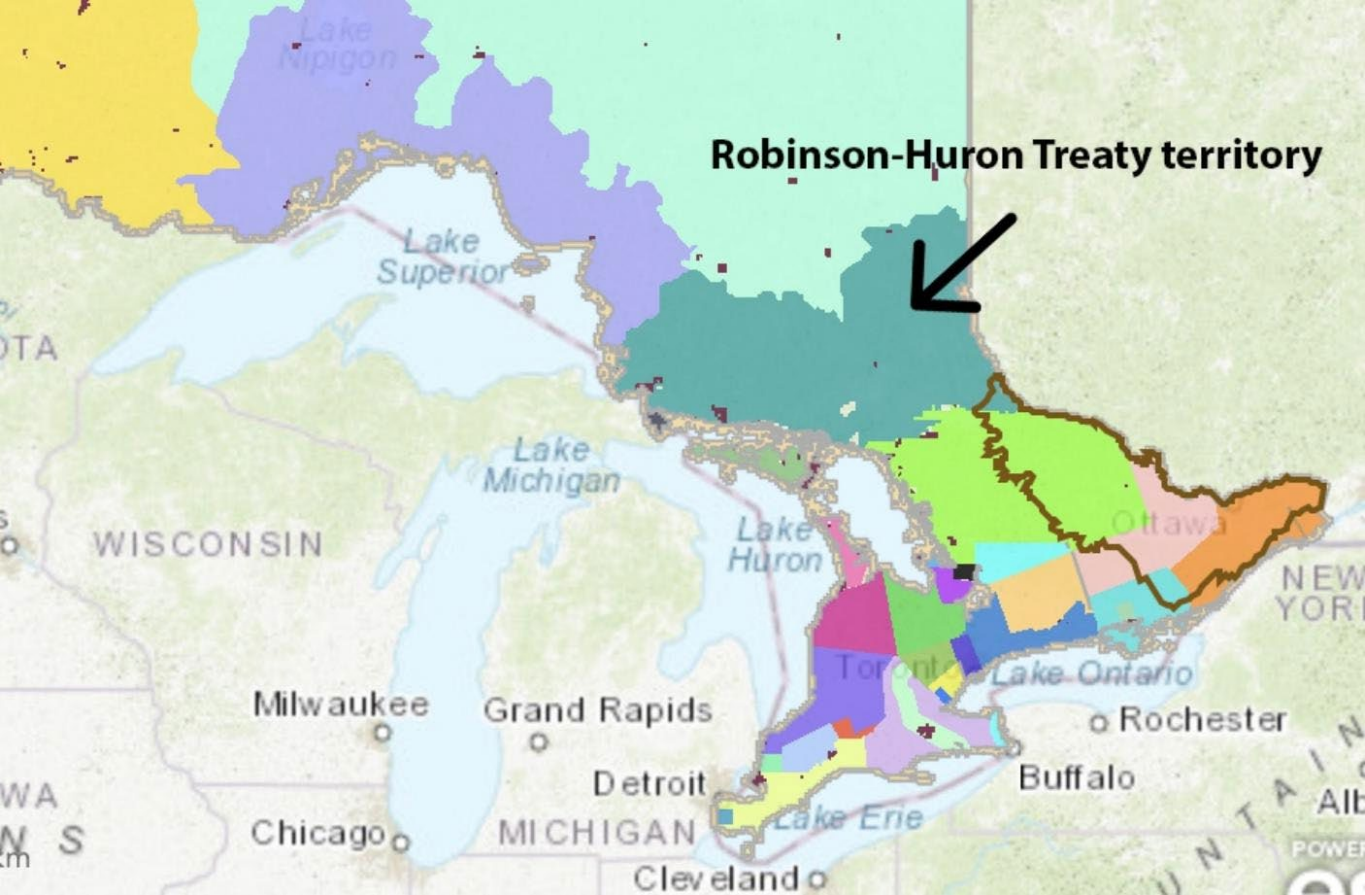
Robinson-Huron Treaty territory

Ketegaunseebee is one example of an Indigenous community within the Anishinaabek Nation and of the great linguistic and cultural diversity in Indigenous Nations and communities across Turtle Island (Siebens & Julian, 2011; Statistics Canada, 2017). The Anishinabek Nation is a “political advocate for 39 member First Nations across Ontario…[it] is the oldest political organization in Ontario and can trace its roots back to the Confederacy of Three Fires, which existed long before European contact” (Anishinabek Nation, 2020, para. 1). Given the vast diversity of Indigenous communities across Turtle Island, a 10-year territorial language strategy in Robertson Huron Treaty territory (see Fig. 4 below) was suggested in our LRC (S. Chiblow, 2021, personal communication) as one way in which CBLP could be developed into broader, wider-ranging culturally and environmentally responsive LPP and self-determined Anishinaabe language education policy. Territorial language strategies have implications for the implementation of the United Nations Declaration on the Rights of Indigenous Peoples (UNDRIP). In Canada, UNDRIP received Royal Assent on 21 June 2021. The UNDRIP Act ensures Canadian federal laws reflect the standards set out in the Declaration, while also respecting Aboriginal and Treaty rights recognized and affirmed in the Canadian Constitution. While one of the main goals of UNDRIP is to support Indigenous Peoples’ right to self-determination, its provisions are non-binding for all levels of government and allow nation-states to adopt a “minimalist approach” (Tollefson & Tsui, 2004, p. 6). For example, *Bill C-91: An Act respecting Indigenous Languages*, which came into force in 2019 to support the efforts of Indigenous peoples to reclaim, revitalize and strengthen Indigenous languages in Canada, “amounts to nothing more than an aspirational policy statement… with no specific Indigenous language rights and no corresponding positive obligations on the Government to implement those rights” (Fontaine et al., 2019, p. 3). And more recently, in Québec, *Bill 96: An Act respecting French, the official and common language of Québec* received assent and became law on June 1 2022. Bill 96 forbids provincial government agencies, municipalities, and municipal bodies in Québec from making use of languages other than French and damages reconciliation efforts with Indigenous Peoples (Serebrin, 2022). A territorial language strategy for Robinson Huron Treaty territory could inform, facilitate, and enable the enactment of a dynamic “territorial language principle” decided by the Anishinabek Nations of that territory and its language dynamics (Kymlicka, 2001; Morales-Gálvez, De Schutter, & Stojanović, 2022). A territorial language principle “grants language rights that are limited to a particular territory in order to ensure the maintenance of a particular language in that area” (May, 2018, p. 246). A dynamic territorial language principle—informed by a territorial language strategy such as that identified as part of our LRC and CBLP using *TEK-nology*—can inform more equitable self-determined language education, planning, policy, and legislation for Indigenous Peoples’ (linguistic) human rights and the implementation of the UNDRIP Act in Canada, currently in its Action Plan stage until mid 2023.

### Strength-based and self-determined community-based language planning

Transmission of conversational knowledge is crucial for ALRR and ILR (Assembly of First Nations, 2019). An important study with Indigenous communities in British Colombia reported that, “youth suicide rates effectively dropped to zero in those few communities in which at least half the band members reported a conversational knowledge of their own “Native” language” (Hallett et al., 2007, p. 1). The analysis indicates that the CBLP *TEK-nology* videos can support learning and intergenerational transmission of conversational Anishinaabemowin among community participants and beyond. The *TEK-nology* videos can enable the fluent Elder to reach more people and foster accessible communities of practice on- and off- reserve (Toth et al., 2018).

Participants stressed the strength of the connections between Anishinaabemowin, culture, community, and identity. Monolingual and monocultural English education is not sufficient (K. Bell, 2021, personal communication). Due to the devastating and ongoing impacts of colonization, such as residential schools, reclaiming an Indigenous language can be a traumatic and complex process (Wesley-Esquimaux & Smolewski, 2004). ALRR is a means to “strengthen one’s identity…[and] get a piece of yourself back” (B. Nolan, 2021, personal communication). Participants also commented that teachings in the language come from the land. The land is “a data source, a teacher, and spiritual guide” (McGregor et al., 2018, p. 116). Phyak & De Costa (2021) underscore, “Indigenous language education extends beyond language-centric perspectives and includes the reclamation of identity, power, and epistemologies of self-determination” (p. 293). The participants on the *TEK-nology* CBLP project demonstrate that there are self-determined community- and context- specific factors that can support ALRR, language transmission, motivation, and progress. I define these factors as *strength-based language indicators*. *Strength-based language indicators* go beyond mainstream standardized tests, linguicentrism (Spolsky, 2014), or dominant language categorizations, such as fluent, intermediate, and/or beginner, to include strength-based processes, such as cultural reclamation, language and community pride, and conversational knowledge (Hallett et al., 2007; Leonard, 2012, 2017; Truth and Reconciliation Commission of Canada, 2015). Participants indicated, for example, that listening to, or understanding Anishinaabemowin is a motivating and important factor (S. Chiblow, 2021, personal communication) and that progress and deeper understanding can take place “without having to be grammatically correct” (S. Nolan, 2021, personal communication). Elder Barbara stressed that “I don’t force them to speak the language, they will speak the language when they are comfortable” (B. Nolan, 2021, personal communication). Wesley Leonard’s (2017) language reclamation framework emphasizes the “cultural, historical, ecological, and spiritual contexts that underlie the way a community defines its language” (p. 17). Language reclamation, and ALRR, “is thus a type of decolonization” (Leonard, 2017, p. 18).

The implications of the CBLP *TEK-nology* pilot project are important for enacting more equitable LPP to address historical and structural inequalities (Tollefson, 1991), “cognitive imperialism” (Battiste, 2013), and “epistemological racisms” (Kubota, 2020). In the Canadian context, the Truth and Reconciliation Commission Report (2015) lists 94 Calls to Action to redress the legacy of residential schools and advance reconciliation. The report stresses the need for mainstream educational reform and increased governmental funding for long-term, community-led and -based ILR initiatives, such as CBLP. As highlighted previously, Bill C-91 does not meet the challenge of creating more initiatives that are controlled by the Indigenous language communities they deem to serve. CBLP using *TEK-nology* can help address this imbalance and inform more equitable LPP. At a home, family, and community micro- and meso- level, the *TEK-nology* pilot project could serve as a model for more self-determined CBLP initiatives in Turtle Island and across the globe during the current United Nations International Decade of Indigenous Languages (2022–2032) and beyond. At a macro-level, majority language planning and policy decision makers, such as in the case of English or French in the Canadian context, could adopt and fund more community-based or territory-based language planning initiatives, such as the *TEK-nology* pilot project, to foster more ethical, pluralistic, and equitable nation-state language policies (May, 2018). Corntassel (2008) underscores, for “substantive decolonization and community regeneration to take place. The identification and implementation of nonstate, community-based solutions should take precedence” (p. 121). The community-led, culturally-grounded, context-specific strategies, pedagogies, approaches, creations, and recommendations identified as part of the CBLP *TEK-nology* pilot project can:

(1) inform future policy and legislation decisions at a territorial, provincial, federal, and national level;

(2) enable full implementation of UNDRIP;

(3) improve relationships with nation-states; and

(4) ensure the full recognition of the inherent rights of Indigenous Peoples, including Treaty rights.

### Limitations and future directions

More research and funding are required to develop the *TEK-nology* approach and the videos on a larger scale. Some respondents on the online survey remarked that more videos could be made, with more content and activities for diverse age ranges. The project relied on one fluent Elder to lend her expertise as one of the few language and knowledge keepers. The process of creating immersive videos takes significant effort and time, from scripting, coaching learner-speaker participants, to filming, recording, translating, and transcribing. Further research would require more funding going forward to ensure the Elder and the process are appropriately supported.

Elder Barbara suggested in our last focus group that we apply as an ad-hoc committee for funding to carry out a year-long project to further support community-led and -based ALRR. This funding could enable us to hire a community-based team to support and finance equipment, translations and transcriptions, video making, and more in creating immersive Anishinaabemowin learning videos based on traditional seasonal activities in Ketegaunseebee and the surrounding territory. Building on the CBLP *TEK-nology* pilot project in this way, by branching out more into the wider community over a longer period of time, could help strengthen inter- and intra- community capacity and support ALRR at a territorial level.

## Conclusion

The *TEK-nology* pilot project is an example of Indigenous community-based language planning (CBLP) conducted entirely through digital and online technologies with implications for status and acquisition planning. A group of family and family friends designed and co-created a series of language learning videos for Anishinaabemowin language reclamation and revitalization (ALRR) from scratch during the COVID-19 pandemic. The *TEK-nology* pilot project demonstrates how Indigenous-led, praxis-driven CBLP, using a technology-enabled Indigenous language acquisition approach, can support ALRR, more equitable language planning and policy (Phyak, 2021), and “culturally grounded contexts of praxis” (May, 2021).

## Footnotes

1 This research, File no. 20-11-048, was approved by McGill University Research Ethics Board.

2 All participants signed consent forms. Their names are used with permission.
